# Facilitations and Hurdles of Genetic Testing in Neuromuscular Disorders

**DOI:** 10.3390/diagnostics11040701

**Published:** 2021-04-14

**Authors:** Andrea Barp, Lorena Mosca, Valeria Ada Sansone

**Affiliations:** 1 The NEMO Clinical Center in Milan, Neurorehabilitation Unit, University of Milan, Piazza Ospedale Maggiore 3, 20162 Milano, Italy; valeria.sansone@centrocliniconemo.it; 2 Medical Genetics Unit, ASST Grande Ospedale Metropolitano Niguarda, Piazza Ospedale Maggiore 3, 20162 Milano, Italy; lorena.mosca@ospedaleniguarda.it

**Keywords:** neuromuscular disease, genetic testing, next generation sequencing, whole exome sequencing

## Abstract

Neuromuscular disorders (NMDs) comprise a heterogeneous group of disorders that affect about one in every thousand individuals worldwide. The vast majority of NMDs has a genetic cause, with about 600 genes already identified. Application of genetic testing in NMDs can be useful for several reasons: correct diagnostic definition of a proband, extensive familial counselling to identify subjects at risk, and prenatal diagnosis to prevent the recurrence of the disease; furthermore, identification of specific genetic mutations still remains mandatory in some cases for clinical trial enrollment where new gene therapies are now approaching. Even though genetic analysis is catching on in the neuromuscular field, pitfalls and hurdles still remain and they should be taken into account by clinicians, as for example the use of next generation sequencing (NGS) where many single nucleotide variants of “unknown significance” can emerge, complicating the correct interpretation of genotype-phenotype relationship. Finally, when all efforts in terms of molecular analysis have been carried on, a portion of patients affected by NMDs still remain “not genetically defined”. In the present review we analyze the evolution of genetic techniques, from Sanger sequencing to NGS, and we discuss “facilitations and hurdles” of genetic testing which must always be balanced by clinicians, in order to ensure a correct diagnostic definition, but taking always into account the benefit that the patient could obtain especially in terms of “therapeutic offer”.

## 1. Introduction

Neuromuscular disorders (NMDs) comprise a clinically and genetically heterogeneous group of disorders that affect about one in every thousand individuals worldwide [1], representing a significant health burden to society. Skeletal muscle (muscular dystrophies, myotonic dystrophies type 1 and 2 (DM1 and DM2), congenital DM (CDM), congenital myopathies (CMs) and metabolic myopathies), skeletal muscle voltage-gated ion channels (periodic paralysis, congenital myotonia), neuromuscular junctions (myasthenic syndromes), nerves/motor neurons (Charcot–Marie–Tooth neuropathies (CMTs), amyotrophic lateral sclerosis (ALS), hereditary spastic paraplegias (HSPs) and spinal muscular atrophies (SMA)) can be primarily affected. Onset may occur at birth (SMA, CDM, CMDs, Pompe disease), during childhood (Duchenne muscular dystrophy (DMD), and many CMs, congenital muscular dystrophies (CMDs)), in adulthood (DM1/2, facioscapulohumeral dystrophy (FSHD). Some limb-girdle muscular dystrophies (LGMDs) and other muscular dystrophies) or have a predominant late-onset (ALS). Progression also varies amongst the different types, and amongst patients: it can be rapidly progressive since birth (e.g., SMA type 1) or even if onset is later in life (e.g., ALS with bulbar onset), or it may be slower over time (e.g., SMA type 3, LGMDs, FSHD, DM2, or hypokalemic periodic paralysis (HOP)) [2].

## 2. The Complexity of Diagnosing a Neuromuscular Disorder

Although NMDs are unique and the clinical presentation varies, they all share some common features: muscle weakness and wasting, often fasciculations, cramps, or muscle pain, and not uncommonly—symptoms of bulbar involvement like respiratory and swallowing problems and cranial nerve palsies [3]. There may be a significant phenotypic overlap amongst the different types of NMDs [4]. Moreover, this heterogeneous neuromuscular picture is often “complicated” by the fact that, in some patients disease penetrance is reduced, onset is variable just as is expressivity [5], and many patients may have predominantly extra-muscular symptoms as part of their disease. This in part accounts for the diagnostic delay, which is known to characterize many of these diseases. Several specialists and professionals may come into play at the time of the initial symptoms and there may be the need for many medical investigations, such as extensive biochemical blood tests, muscle magnetic resonance imaging (MRI) or other imaging techniques, neurophysiological assessments, muscle and/or nerve biopsies, lumbar puncture and other diagnostic tests [6,7]. Table 1 summarizes the multiple clinical presentations of the most frequent NMDs and the possible time-lag between initial symptoms and the clinical or genetic confirmation of disease.

The vast majority of NMDs has a genetic cause, with about 600 genes already identified (see http://www.musclegenetable.fr/index.html, accessed date: 13 April 2021), and this number is still growing; pathogenic variants involved display autosomal recessive, autosomal dominant or X-linked inheritance [1] as well as mitochondrial inheritance. For different NMDs, many genes are involved (genetic heterogeneity) and a great variety of mutation types can be found in a single gene (allelic heterogeneity). The full mutational spectrum reported in NMDs includes single nucleotide variants, large deletions and duplications, small mutations, expansion repeats, epigenetic changes, dynamic mutations and atypical mutations or alterations occurring in regulatory regions as promoters, untranslated 5′/3′ regions, or intergenic segments [14]. While, on one hand, genetics facilitates the diagnostic process, it adds also complexity. Not infrequently, the family history is reported to be negative, or genetic testing in the family members or parents is inconclusive. In these cases, a de novo mutation should be considered, along with a somatic mosaicism in which a mutation may be present in some, but not all cells [15]. Moreover, despite the progress in genetics, there are still a number of patients with a probable NMD based on the clinical and laboratory data (e.g., neurophysiological studies and muscle biopsy results) in whom there is no genetic confirmation [16,17].

## 3. The Approach to Genetic Testing

Due to the significant costs of most molecular tests, in terms of both human resources and reagents, it is crucial to establish as precise a clinical diagnosis as possible. The most important step is to consider if the patient’s symptoms may have a genetic origin. There are some features which can suggest a hereditary process: longstanding or slowly progressive deficits, clinical signs out of proportion to the patients’ symptoms, early onset of them, similar symptoms reported in other family members, and the association with musculoskeletal abnormalities, such as pes cavus, scoliosis or contractures. Sometimes patients are unable to identify slowly progressive deficit or recognize similar symptoms in other family members, particularly if they have not received a confirmed diagnosis. Specific questions regarding early milestones, participation in sports, or other physically demanding activity is often necessary to reveal subtle deficits in neuromuscular function [15]. When clinicians have considered the possibility of a NMD, the second step is to localize the disease process (muscle, neuromuscular junction, peripheral nerve or motor neuron). In such a way, ancillary tests like neurophysiological testing, laboratory testing or muscle biopsy may be required to exclude other acquired disorders and narrow the differential diagnosis to allow for targeted molecular testing. Despite these measures, the diagnostic yield of neurogenetic testing can be low even if multiple tests are pursued. Table 2 describes some of the most common signs or symptoms, which may help clinicians to localize the site of lesion and better target the subsequent work up, including genetic testing.

## 4. The Evolution of Genetic Techniques and Their Application to NMDs

**Figure 1:**
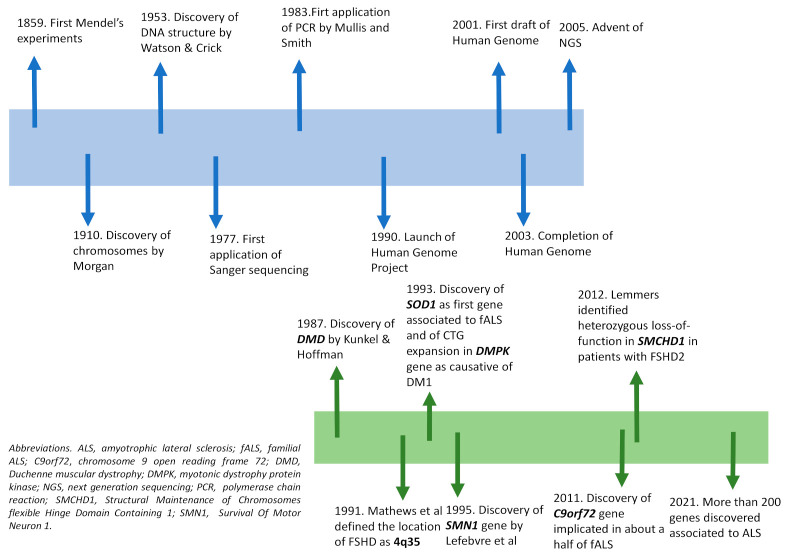
Timeline representing the main genetic discoveries (**top**) and the main genes discovered in Neuromuscular disorders (NMDs) (**below**).

The scientific history of genetics began with the introduction of the fundamental laws of inheritance by Mendel in 1859, and was improved in 1910 by Morgan’s experiments, which revealed that genes were responsible for the appearance of a specific phenotype located on chromosomes [18]. In 1953, Watson and Crick described the structure of DNA and showed that genetic information is represented by a sequence of nucleotides on its two strands [19]. The genetic code was finally uncovered in 1966, by defining that a sequence of adjacent three nucleotides (codon) codes for amino acids. All such findings brought a rapid improvement to the genetics field and to the development of new molecular technologies. The first genetic analysis was performed in the cytogenetics field, making possible the identification of a number of structure abnormalities of human chromosomes [18]. The detection of single nucleotides changes in DNA was instead rapidly developed after the setting-up of polymerase chain reaction (PCR) by Mullis and Smith in 1983, enabling the generation of thousands to millions of copies of a particular DNA sequence [20]. At first, PCR was applied to techniques widely used for known mutations screening. The need to detect every genetic variant was overcome by the introduction of chemical sequencing technology; in particular, the development in 1977 of the dideoxynucleotide chain termination sequencing by Sanger enabled DNA reading at base pair resolution. Quickly, thanks to the introduction of automated DNA sequencers, the manual method was improved and replaced by the automated one [18,21]. All such technological advances were useful in launching of the Human Genome Project in 1990; the draft of the human genome, first released in 2001, was then completed in 2003, leading to the release of the sequence of the entire human genome, the illustration of the vast genetic diversity in humans, and the identification of a large number of disease genes [6,22]. Such a project also contributed to the improvement of sequencing technology, up to the development in 2005 of next generation sequencing (NGS). In contrast to Sanger sequencing, which involves reading of contiguous piece of DNA 1 base at time, NGS utilizes massively parallel sequencing to generate millions of short reads (100–200 base pairs each) at once, which are then aligned to a reference sequence (Figure 1).

Depending on the extent of genetic sequences to be analyzed, testing may be designed to sequence a set of genes associated with clinically related syndromes (gene panel sequencing, GPS), the protein encoding regions of the genome (whole-exome sequencing, WES), or even the whole genome of a patient (whole-genome sequencing, WGS) [23]; the method, therefore, makes possible the screening of many genes/genomic regions simultaneously, in a far more cost- and time-effective manner. Concerning NMDs, there are still examples where single gene testing (e.g., Sanger sequencing, multiple ligation probe analysis-MLPA) should be considered as a standard and first test; this is especially true if the majority of disease causing mutations for a given disease entity are quantitative rather than qualitative (e.g., DMD or SMA), or if the pathology of interest is caused by a single gene (monogenic) or by repeat expansions (e.g., spinocerebellar ataxias, SCAs) [24]. Certainly, NGS has revolutionized the diagnostic approach of many NMDs, being the most commonly used method in clinical practice for first-line diagnosis of diseases for which a wide range of genetic aberrations might be responsible for a similar phenotype, including congenital muscular dystrophies and congenital myopathies, limb girdle muscular dystrophies, congenital myasthenic syndromes, hereditary neuropathies, mitochondrial myopathies and motor neuron diseases such as ALS [25]. Additionally, allowing a better depth and coverage of gene, NGS improves discovery power by identifying novel gene variants not previously associated with a disease [7]; in nine years, NGS has resulted in a near doubling of the number of genes implicated in NMDs, from 290 in 2010 to 535 in 2019 [25]. One typical example is represented by ALS, whose field continues to develop rapidly with multiple disease gene discoveries per year. Ten years ago, its commercial genetic testing was limited to sequencing of SOD1, the first ALS-associated gene identified in 1993 [26]; actually, about 200 genes have been discovered as associated to this pathology [27], with a consequent obvious relevance for diagnosis and genetic counselling.

## 5. NGS and Its Hurdles

With the advent of NGS approaches a growing number of causative variants can be identified [28,29,30]. Even so, the majority of patients with NMDs still remain undiagnosed with variable success rates, mainly depending on the selected patient population and the applied method [31,32,33,34,35,36,37,38,39]. It is, therefore, a major challenge facing clinicians and geneticists to further enhance the application of NGS techniques. For example, it is a subject of ongoing debate which exact NGS approach is optimal from a diagnostic and cost-point perspective [40].

Detailed phenotyping obtained from a complete and accurate clinical evaluation is certainly important to begin the diagnostic work-up and it is increasingly recognized as a prerequisite for NGS-based diagnostics and research. In addition, the effective use of NGS in diagnostics, regardless of the approach chosen (GPS, WES or WGS), should take into account information regarding the workflows relevance, such as analysis, coverage and sequencing depth to understand each specific clinical application and diagnostic capabilities.

All NGS approaches, even GPS, generate a large volume of sequencing data which have to be processed by proper bioinformatics pipelines: the larger the genomic region to investigate (from GPS to WGS), the smaller the average sequence depth [41], and the greater the number of variants identified. Analysis of such sequencing data requires an important computational effort and needs skilled bioinformaticians able to use and choose the different tools available in each sequencing analysis step [42].

### 5.1. GPS Panel Sequencing

GPS test consists of multiple genes sequenced at the same time and secures that all coding exons of the genes of interest are targeted and sufficiently high covered; the majority of panels are probably custom-made, although for some more common diseases, commercially panels are available; both custom-made panels can include a single very long gene up to several hundreds genes of interest. Genes usually are grouped together based on producing the same phenotype when mutated, and for such reasons, the procedure is especially indicated as a first-tier diagnostic method if clinical diagnosis of a heterogeneous disorder does not lead to a particular gene [24]. GPS are frequently used in routine diagnostics since are cheaper then WES and WGS due to fewer genes targeted and require less data processing, analysis and storage. Since the analyzed region is smaller, deeper coverage is obtained, allowing a better detection of some copy number variations (CNVs) (e.g., PMP22 duplication/deletion [43]) and mosaicism, compared to WES [44]. In addition GPS do not reveal findings unrelated to the phenotype being investigated, avoiding incidental findings and ethical problems [44].

While these genomic tools are not capable of isolating genes associated with novel diseases, they are successfully used in the field of clinical diagnosis of NMDs [45,46], especially of those characterized by clinical overlap and oligogenic inheritance. For example, NGS panel of 56 putative candidate genes codifying for proteins involved in excitability, excitation-contraction coupling, and metabolism of muscle fibers has been demonstrated to be a useful approach in the molecular diagnosis of skeletal muscle channelopathies [47]. Moreover, in an Italian study focused on molecular analysis of familial ALS patients, the detection rate of pathogenic variants using GPS (45%) was higher respect to Sanger sequencing (23.8%), due to the mutations found in minor ALS genes [48], thus demonstrating the usefulness of targeted sequencing in ALS molecular diagnostics.

The biggest challenge of a gene panel for a given disease consists in its design; attention should be paid to which genes to include in order to maximize the diagnostic yield, and simultaneously minimize costs and volume of sequencing data obtained. A periodic update of the genes list in panels is needed, due to the frequent and continuous identification of novel causative genes.

### 5.2. Whole-Exome Sequencing (WES)

WES is able to encompass the entire coding regions of the genome where an estimated 85% of disease-causing variants are believed to occur [3]; it is often performed in unsolved cases after a GPS approach, in patients affected by unknown diseases [4] or in cases where no reasonable hypothesis about which gene is causing the NMD can be made [7]. Therefore, WES has the inherent potential to identify novel disease genes and allows a diagnostic re-evaluation at a later time.

Concerning the isolation of disease-causing genes, two main approaches are usually used. The first consists in the analysis of WES (and WGS) of a group of patients characterized by the same clinical features and consecutive filtering of variants located in a common gene for all or some of the members of the studied group. The second one is represented by the analysis of isolated patients in conjunction with parents (trio analysis) and/or informative members of their family, and filtering of variants by different mode of inheritance [44].

The first proof-of-principle study for exome sequencing in NMD was performed for Charcot–Marie–Tooth neuropathies: WES was applied in a large family and a causative mutation in GJB1 was identified in two affected individuals [49].

Over time, the diagnostic value of WES in NMDs has been demonstrated in several studies. Haskell et al. (2018) performed WES in 93 NMDs pediatric and adult patients with overall diagnostic yield of 12.9%, and only 63% prior phenotyping testing, including invasive muscle biopsy, was informative to reach the diagnosis [39]. Waldrop et al. (2019) performed trios-WES in 31 pediatric patients yielding a diagnostic rate of 39%; two rare genetic cases, Vici syndrome associated with EGP5, infantile hypotonia with psychomotor retardation, and characteristic facies—three caused by TBCK pathogenic variants, were identified. With positive genetic diagnosis and proper surveillance, treatment could be provided [50]. The diagnostic utility of comprehensive GPS and WES has been considered to be comparable in practice [24,51]. In contrast, it is still unclear whether the widely used small-scale panels, as often mandated by national health care providers, achieve similar results [40,50]. Another issue requiring refinement is the correct identification of causative variants against the abundance of irrelevant background variation. The widely used guidelines of the American College of Medical Genetics and Genomics (ACMG) consider various strands of genetic and clinical evidence for variant classification [52]. Whilst some variants can reliably be classified as benign or pathogenic right away, the causative effect often remains uncertain after genetic testing (variants of unknown significance, VUSs) [53]. It has already been shown that uncertain findings can be successfully reclassified using clinical reconsideration, complementary family genotyping or supporting functional data [54,55,56]. Such approaches have the ability to reveal minor and initially overlooked clinical features, bringing to light specific phenotypic fits potentially underpinning the pathogenic relevance of variants. The WES approach was also able to discover a wide range of phenotypes associated with some disease genes, finding a connection between what had previously considered distinct clinical entities. In congenital myopathies, the traditional classification based on histopathological findings is now flanked by genetic classification [57]. For example, the term “congenital titinopathy” is now suggested to describe a group of titin (TTN)-related diseases [58], as the term “ryanodine receptor (RYR)-related myopathies” similarly includes a wide phenotypic range [59]. Although WES is considered a powerful tool in molecular diagnostics, it suffers from some limitations: short-read WES is of limited usefulness for detecting variants other than single nucleotide variants (SNVs) and small insertions/deletions (indels), such CNVs, expansions, or contractions in repetitive regions, chromosomal rearrangements and deep intronic variants. CNVs such as exon deletion in SMN1 in SMA, exon deletion or duplication in dystrophinopathy, PMP22 duplication in Charcot–Marie–Tooth diseases could be evaluated by MLPA, specific GPS or WGS. Expansion or contraction in repetitive regions including CTG triplet repeats in DM and contraction of the D4Z4 macrosatellite repeat in DUX4 in FSHD could be evaluated by fragment analysis. Correct clinical diagnosis of these distinctive NMDs guiding the appropriate target gene study would avoid unnecessary WES that could not detect these variants. WES may also miss the variants outside the exome that arise in the deep intronic or untranslated regions (UTR); it is estimated that 15% of variants potentially causative of mendelian traits are localized in non-coding regions of the genome and all these variants would be missed performing WES [60].

### 5.3. Whole-Genome Sequencing (WGS)

The limitations discussed above can be overcomed by the use of WGS; this approach is characterized by an uniform coverage in coding and non-coding regions and is able to detect CNVs, gross chromosomal abnormalities and deep intronic variants [4]. WGS represents a powerful tool for genomic research, since it may solve WES-negative results obtained in patients affected by a NMD.

In the neurogenetics field, WGS was first successfully applied to a recessive form of CMT disease with an unknown genetic basis: thanks to this approach, variants in the novel SH3TC2 associated gene were identified and a genetics diagnosis was made [61].

In literature, there are some other examples of NMDs diagnosed with WGS. Such approach identified truncating mutations in RBCK gene in a family quartet with two children, both affected with a previously unreported disease, characterized by progressive muscular weakness and cardiomyopathy [62]. Recently, a novel insertion in PMP22 gene was linked with a clinical diagnosis of CMT3 thanks to WGS, supporting the heterogeneity of PMP22 related to CMT [63].

**Figure 2:**
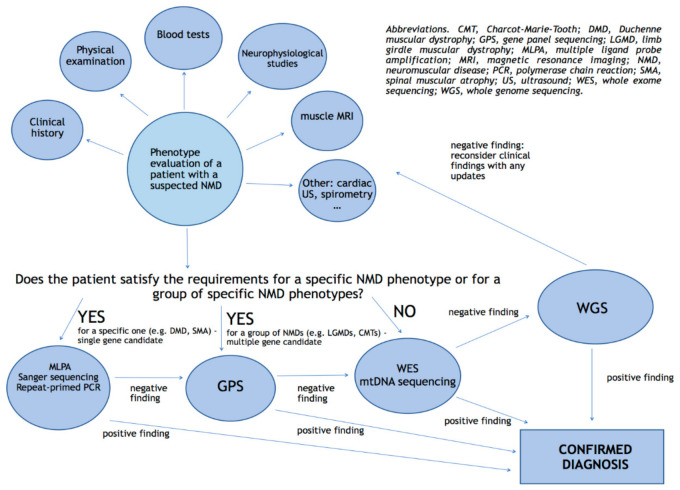
Proposal for a diagnostic algorithm of genetic testing in NMDs.

Rapid WGS is a faster approach of NGS which can return results in as little as 26 h with high precision and sensitivity. Usually, analysis is focused on ~6000 genes causative of the known monogenic disorders, and is further limited to variants in genes that ranked high in correspondence to the phenotype of the affected infant/child. If a single, likely causative variant is identified for an autosomal recessive condition, the entire coding region is manually inspected [64]. Often, rapid WGS of parent–infant trios are conducted since the approach is critical for recognition of de novo variants. Petrikin et al. (2015) applied a rapid WGS approach to a select a population of ill infants in a Level IV neonatal intensive care unit (*n* = 35), reaching a diagnosis of a causative genetic disease in 57% of patients (20% of neurological findings). Moreover, since WGS also provides good coverage of the mitochondrial genome, one maternally inherited diagnosis in the 35 cases was obtained [64]. The major limits in using WGS today in daily routine diagnostics consist in costs and interpretation: computational infrastructures suited to store and analyze terabytes of data are necessary, as well as experience in variant interpretation [3,4]. In addition, since WGS reveals about 3 to 5 million variants per individual [65], it may also return incidental findings that may be relevant to the patients current or future health yet unrelated to the initial line of questioning. Moreover, a study conducted by Alfares et al. (2018) reported that diagnostic yield from WGS was only 7% higher than WES, recommending the reanalysis of WES raw data before performing WGS [66] (Figure 2).

### 5.4. Mitochondrial Genome Sequencing

The clinical diagnosis of mitochondrial disorders has always been challenging. Although several well-defined clinical syndromes are easily recognized (such as chronic progressive external ophthalmoplegia, CPEO; and mitochondrial encephalomyopathy with lactic acidosis and stroke-like episodes, MELAS), many patients or families do not manifest all the canonical symptoms and signs; so this clinical heterogeneity, together with the vast genetic heterogeneity, often makes the diagnosis of mitochondrial diseases difficult [67].

The genetic basis of mitochondrial disorders is indeed complex: the mitochondrial proteome shows a dual genetic origin and therefore pathogenetic variants can reside in both nuclear and mitochondrial DNA. Moreover, any mode of inheritance (maternal, autosomal recessive, autosomal dominant, and X-linked) are described and can lead to both familial and sporadic cases. However, the majority of adult patients with mitochondrial diseases have mutations in mitochondrial DNA (mtDNA). Pathogenic deletions or SNVs of mtDNA usually affect a proportion of mtDNA molecules (heteroplasmy) [67]. Since the first discovery of mitochondrial disease-causing variant in the mtDNA in 1988 [68], technologies for genetic testing have evolved from the targeted mtDNA and candidate gene Sanger sequencing, to the more unbiased and systematic technologies based on the NGS. Although candidate gene and mtDNA sequencing remain fast and cost-effective methods for genetically and phenotypically well-defined syndromes, such as the Leber’s hereditary optic neuropathy (LHON), the genetic heterogeneity of mitochondrial disorders, together with often unspeficic biochemical and metabolic findings, makes the choice of feasible number of candidate genes difficult. Indeed, screening of 64 candidate genes through Sanger sequencing established a diagnosis in just 11% of cases [69].

The use of NGS-based approaches has enabled analysis of nuclear genes simultaneously with mtDNA. WES particurarly has been successfully used to detect both nuclear and mtDNA variants in mitochondrial disorders. Given the cost constraints and additional complexity of WES, it is more commonly used only after obtaining negative results from targeted analysis such as mtDNA sequencing.

On the other hand, the NGS era caused a revolution in genetics of mitochondrial disease. Apart from diagnostic rates and expanding the genotype-phenotype association, it accelerated discovery of novel disease genes, which is over 20 per year since 2012 [70]. Starting with the more targeted approaches, application of NGS to sequence mtDNA is a routine first step in many diagnostic centers, especially for the cases with adult onset and where phenotype is highly evocative of a mtDNA etiology [71]. Apart form providing variant discovery, it also allows exact measurement of heteroplasmy levels [72]. Considering that in pediatric-onset cases, analysis is usually performed in urine and blood, instead in adult-onset ones it is usually performed in muscle, as the affected tissue is the most informative and causative variants may be undetected in blood due to tissue-heteroplasmy. In fact, as observed in CPEO, single large-scale mtDNA deletions are mostly affecting the post-mitotic skeletal muscle.

Expanding the diagnostic focus to the nuclear genes, GPS provide a targeted, deep sequencing of the predefined sets of mitochondrial disease genes, as well as candidate genes encoding for the proteins involved in essential mitochondrial function, whose disruption is thus likely to cause a disease. Available panels range from 100 genes associated with complex I efficiency to the “MitoExome”, targeting the predicted mitochondrial proteome: the success rate varies from 7% to 31% [73,74,75,76]. GPS offer advantages in the higher coverage of targeted regions, as well as easier data interpretation; however the constant updates of reported disease genes, the often low phenotype–genotype correlation, the inability to surely define a mitochondrial disease by clinical symptoms, and the lower diagnostic yield of GPS compared to WES have made the latter the more preferable choice [71]. In modern diagnostics, WES has become a desired first-tier tool of investigation, especially in the cases of early-onset mitochondrial disease, where the cause of disease likely lies in the nuclear DNA [77] and because it also allows the analysis of mtDNA in the given tissue [78]. Within rare disease-diagnostic cohorts, mitochondrial diseases sit at the upper end of the WES diagnostic rate [79], ranging from 35% to 70% [80,81,82].

Limitations of WES regarding the genome coverage can be overcome with whole genome sequencing (WGS). Recently, trio-WGS was performed in an Australian cohort of 40 pediatric patients with clinical features suggestive of mitochondrial disease reaching a definitive molecular diagnosis in 55% of cases; moreover, three potential novel genes (ARX, NBAS and SKIV2L) associated to mitochondrial disease were identified [83].

### 5.5. Data Analysis and Challenges

Despite its enormous strengths and potentialities, NGS has also limitations and challenges, especially in the diagnostic field in which reaching a molecular diagnosis is fundamental: troubles regard especially the bioinformatic analysis and data interpretation.

NGS needs a bioinformatic workflow which is extremely complex: output signals generated by the NGS platform are converted in short sequences of nucleotides (short reads, ≈100–200 bp) to which base quality scores are then assigned. Reads are aligned to the reference genome and genetic variants are called, filtered and then subjected to interpretation: this step is more and more difficult going to increase the extension of the analyzed genomic regions.

Computational algorithms are used at all stages (alignment, variant calling, annotation, interpretation) and are still subject to final optimization. Different software packages are available and may result in different final interpretations; the use of different thresholds for statistical significance and variant calling would produce a different final list of putative genes.

In a typical pipeline, raw sequence data are aligned to the reference sequence using an aligner software, with the resulting alignments typically store in binary alignment map (BAM) file format; BAM files represent the standard format for storing and sharing NGS data. Prior to variant calling, routine quality control of analysis-ready BAMs should be performed with the aim to evaluate key sequencing metrics, verify the achievement of a sufficient coverage and check samples for the possible presence of contamination [41]. Incorrect mapping of reads can readily lead to erroneous identification of sequence variants, highlighting the importance of alignment accuracy; the most common alignment problem arises from reads that map to multiple locations on the reference sequence (multireads) and their correct assignment to the original sites remains challenging and fundamental. For SNVs/indels detection, the choice of a single variant caller is usually sufficient, since their detection tools have demonstrated high accuracy. However, combining the results of different callers, may offer a slight sensitivity advantage; without a “gold standard” calling algorithm, one may focus on those variants that are called by two or more callers to ensure a better chance of validation [84].

NGS, providing horizontal coverage and accuracy rates < 100%, could result in false positive results and missing variants (false negatives). Artifactual variant calls are often related to errors in short-read alignment and can be systematically filtered without significantly compromising sensitivity. For clinically relevant variants, a visual review of the alignment is recommended in order to identify false-positive variant calls that slip past automated filters. Several frequently occurring artifacts that can be identified by manual review are represented by low-quality base calls, read-end artifacts due to local misalignment near indels, strand bias artifacts, erroneous alignments in low-complexity regions and paralogous alignments of reads not well represented in the reference.

Concerning de novo variants, in addition to filtering for artifactual calls, they should be queried against public databases of genome variation, such as the gnomAD database [41].

There is significant debate within the diagnostics community regarding the necessity of confirming NGS variant calls by Sanger sequencing, considering that numerous laboratories report having 100% specificity from the NGS data alone [85]; probably, the burden of additional confirmatory testing is likely to decrease as technologies continue to evolve.

While pipelines have been primarily focused on the removal of false positives, less attention has been paid to the characterization of the fraction of false negatives, whose rate is strongly dependent on calling pipeline parameters, and especially, on read coverage. Since false negatives rate has been shown to be higher (~6–18%) than that of false positives (<3%) [86], missing mutations have to be considered a significant feature of genomic datasets and demand additional fine-tuning of bioinformatics pipelines.

Another critical point of bioinformatic workflow is represented by the variant classification and interpretation, mainly for effect of VUSs. It is incredibly difficult to prove causality for variants never reported, or located in a gene that has never been associated with disease or in a gene previously associated to a different phenotype: functional studies, segregation studies, additional families and other genetic analysis are essential to support the link [44].

A process that today is considered useful for a possible reclassification of previously identified VUSs or, more generally, for an increase in the diagnostic yield of non-diagnostic NGS is represented by the periodic “reanalysis” of archived NGS data: since annually ~250 gene-disease and ~9200 variant-disease associations are reported, this increase in information helps to establish additional diagnoses and maximize the diagnostic performance. Wenger et al. (2017) comprehensively reanalyzed 40 unsolved WES cases for which a nondiagnostic exome report was issued, on average, 20 months before reanalysis; a definitive diagnosis was identified in 10% (4/40) of cases [87] showing that a “negative” nondiagnostic result from NGS sequencing does not always mean that the disease etiology lies outside of the data already produced.

Although notable improvements in molecular analysis and bioinformatics are continually described, the technical limitations of short-read NGS are well known. Approximately 8.5% of the genome is extremely resistant to SNVs/small indels calling due to repetitive sequence or segmental duplications, causing poor variant detection in some clinically relevant genes [44]; this also have an effect in the detection of expansions or variants within NEB and TTN triplicated regions [43]. Moreover, in terms of capture efficiency, an important subset of GC-rich exons of coding genes is missed; accordingly, causative disease mutations present in these regions will be missed. Finally, the presence of highly homologous regions could generate coverage deficiency. Although these regions are captured and covered by multiple reads, quality control filters discard them because the same read can be aligned in multiple different regions, and therefore, coverage drops and variants present in those regions may be missed [44].

To overcome such technical limits, novel sequencing (e.g., long-read sequencing) and informatics are needed to find genetic variants that may be resistant to detection with the current standard NGS procedures.

### 5.6. Emerging Technologies

An innovative research sequencing that could provide opportunities to solve many complex problems linked to short-read NGS is long-read sequencing, also called third-generation technology. It can achieve read lengths as high as 15 kb (average of 3 kb) [88], well beyond Sanger or short-read NGS technologies, and therefore, it enables an improved detection of large indels, structural variations, haplotyping and repeat expansions [89].

Such technology in a research context has been shown to be able to capture clinically relevant variations, such as the D4Z4 repeat expansion responsible for FSHD with an estimated sequence accuracy of the total repeat region of 99.8% based on a comparison with the reference sequence [90].

Several long-read sequencing technologies have been successfully tested also for the detection of the exanucleotide repeat expansion in C9orf72 gene [91,92] which is the most common genetic cause of ALS and frontotemporal dementia (FTD) [93]. The technology endeed can span the entire C9orf72 GGGGCC expansion facilitating reliable estimation of expansion sizes and shows the ability to evaluate sequence content; this might help to determine the presence of interruptions in C9orf72 expansions [91] which is highly relevant since interruptions act as disease modifiers in other repeat expansion disorders [94].

The use of short-read or long-read sequencing depends on the research or clinical application [89]; however, as the technology and bioinformatic tools continue to improve, long-read sequencing will likely become a regular feature in the rare disease genomics tools kit [43].

Despite the tremendous impact, the diagnostic yield of all technologies described is far from complete: short- and long-sequencing enables the detection of very numerous coding and non-coding variants, but equally enormous advances in characterizing especially the non-coding alterations have not been met. RNA-sequencing (RNA-seq, also called transcriptome sequencing) analysis is able to add crucial functional evidence to the genetic information obtained by WES and WGS, and enables an increase in the diagnostic yield of different pathologies.

RNA-seq applies NGS technologies to qualitatively and quantitatively profile the full set of transcripts (transcriptome), including mRNAs, small RNAs and other non-coding RNA [84]. The procedure involves isolation of total RNA from tissues or cells of interest; RNAs are purified, fragmented and reverse transcribed into cDNA molecules which then are enriched by PCR. Following quality control and quantification, libraries are finally subjected to sequencing [65]. Similar to DNA-Seq analysis, RNA-seq data analysis involves base calling, reads mapping, transcriptome reconstruction, and also expression quantification and differential expression analysis [95].

This technique provides an opportunity to evaluate the real effect of the variation in the DNA as it undergoes transcription and is valuable as a complementary diagnostic tool; it not only permits the detection of genetic variants at the mRNA sequence level, but allows direct probing of the effect of genetic variants by assessing altered expression levels, aberrant splicing, or gene fusions [96]. Therefore, observing changes at the mRNA level can point towards the pathogenetic variant that might have otherwise been ignored (e.g., cryptic splice site) or not to be observed with WES or WGS (e.g., large structural change) [65].

RNA-seq analysis is also a useful approach in providing crucial functional evidence for pathological relevance in aberrant splicing of some VUSs or synonimous variants that previously evaded variant prioritization through NGS applied to DNA [65].

RNA-seq approach is widely used in the cancer field for its ability to detect gene fusions [65] and as a prognostic outcome measure, e.g., by assessing the expression of certain gene sets aiding treatment decisions for breast cancer or leukemia, and for monitoring immune responses hinting at possible rejections following organ transplantation [96]. However, different studies reported on RNA-seq performed on NMDs. Cummings et al. (2017) studied with this approach a cohort of 50 patients with NMDs: RNA-seq enabled validation of candidate splice-disrupting mutations and identified splice-altering variants in both exonic and deep intronic regions, yielding an overall diagnosis rate of 35%, and resulting in the discovery of a recurrent de novo intronic mutation in COL6A1 [97] which is now known to be a common cause of collagen VI-related dystrophies [98]. A similar approach applied to patients’ fibroblasts resulted in molecular diagnosis in 5/48 patients (10%) affected by mitochondrial disease previously undiagnosed by WES. This technique detected aberrantly expressed genes, aberrant splicing events, and monoallelically expressed rare variants as the molecular cause in patient-derived fibroblasts, and identified a novel mitochondriopathy disease associated gene (TIMMDC1) [99]. A third study conducted by Gonorazky et al. (2019) used RNA-seq in 25 NGS-negative patients affected by monogenetic NMDs and found a genetic cause in 36% of them; moreover they establish that blood-based RNA-seq is not adequate for neuromuscular diagnostics, whereas myotubes generated by transdifferentiation from fibroblasts accurately reflect the muscle transcriptome and faithfully reveal disease-causing mutations [100]. Taken together, all these studies clearly demonstrate the power of RNA-seq to reliably detect pathogenic RNA defects in NMDs diagnosis that were not evident solely from genetic information.

Potential disease-causing variations in non-coding DNA can be successfully scanned applying NGS to DNA and RNA simultaneously. RNA-seq of leukocytes of a patient with sporadic atypical SMA identified a highly significant and atypical ASAH1 isoform not explained by a missense mutation previously found by DNA sequencing providing a molecular diagnosis of autosomal-recessive SMA with progressive myoclonic epilepsy [101]. Again, a combining WGS and RNAseq analysis was applied to a large consanguineous family in which members displayed autosomal recessively inherited SCA: homozygosity mapping, rare variant search, and comparison of the transcriptomes of affected and unaffected family members led to the detection of a causative homozygous point mutation in non-coding RNA RNU12 [102].

Finally, RNA-seq can also help to determine relative abundance and stability of transcripts that might correlate with disease severity and prognosis [25].

## 6. Discussion

Providing patients with a genetic diagnosis is nowadays mandatory. Diagnosis gives a chance for these patients to be recruited in clinical trials and it also helps in their care. It provides the mode of inheritance and can help define the prognosis, progression, and critical comorbidities for screening [1]. The American Association of Neuromuscular and Electrodiagnostic Medicine (AANEM) recognized the importance of genetic testing in NMDs and produced a consensus statement regarding its clinical utility, pointing out its fundamental role in the diagnosis and management because of cost effectiveness, disease management, quality of life, and family planning [103]. Moreover genetic testing allows access to therapy or enrollment in novel clinical trials or disease registries. This is even more true given the availability of personalized therapies; examples are the new drugs used in SMA [104,105], or the identification of the presence of the C9orf72 hexanucleotide repeat expansion or SOD1 mutations in ALS as a necessary criterion for enrollment into clinical trials for antisense oligonucleotide (ASO) therapy [7]. Establishing a specific molecular diagnosis is important for several reasons: (1) for disease management and treatment; (2) to decrease psychosocial burden because management and prevention protocols may be adopted; (3) to prevent unnecessary treatments and diagnostic procedures for other family members and for the patients in case symptoms may be related to the disease process itself without needing further investigations (e.g., liver biopsies for increase in liver enzymes which are to be interpreted in the muscle disease process itself); (4) to identify recurrence risk and genetic counselling to family planning and (5) to participate in clinical trials and patient registries [106]. Referring physicians should be very clear on the limitations of genetic testing during counselling and the following “points” should be emphasized: (a) a negative result does not exclude a genetic basis or contribution to the condition; (b) the test may be uninformative if a VUS is identified; and (c) positive results do not uniformly allow prediction of penetrance or disease course. Families who are not ready to undergo genetic testing may consider DNA banking to permit future testing [107]. As treatment options become available the approach to genetic testing in children will need to be revisited especially thinking that experience from previous trials and real-world data for example in SMA [108,109] strongly supports and provides evidence that the earlier the treatment, the better the outcome.

## 7. Conclusions

Genetics in neuromuscular disorders is extremely complex. The clinical evaluation is fundamental to target the appropriate genetic testing. A negative result should direct clinicians towards other single gene analysis or towards wider sequencing approach such as GPS, WES and WGS. Uncertain findings (such as VUS) still remain a challenge for clinicians in this “diagnostic odyssey”. Pursuing the genetic diagnosis should always take into account the benefits that the patients can obtain in terms of therapeutic offer or trial enrollment.

## Figures and Tables

**Table 1:** Main neuromuscular conditions and time lag between onset and diagnosis.

Neuromuscular Disease	Common Neuromuscular Presentation	Common Extramuscular Presentation	Time-Lag between Onset of Symptoms and Diagnosis
Duchenne muscular dystrophy (DMD)	Very high CK levels Proximal LL weakness Calves hypertrophy	Intellectual disability/autism	24 months [8]
Spinal muscular atrophy (SMA)	Hypotonia and respiratory failure (if birth onset) Proximal muscle weakness and absent DTRs (if adult onset)	_	4.7 ± 2.82 months (type 1) 15.6 ± 5.88 months (type 2) 4.34 ± 4.01 years (type 3) [9]
Congenital myotonic dystrophy (CDM)	Mixed hypotonia at birth	Intellectual disability Difficulty breathing Swallowing problems Talipes	Few days from birth [10]
Myotonic dystrophy type 1 (DM1)	Hand and foot dorsiflexor weakness Hand myotonia Bilteral ptosis Facial weakness	Early-onset cataracts Cardiac arrhythmias Syncope/cardiac arrest Gonadal failure Insulin resistance Excessive daytime sleepiness	7.3 ± 8.2 years [11]
Myotonic dystrophy type 2 (DM2)	High CK Difficulty climbing stairs Muscle pain	Early-onset cataracts Cardiac arrhythmias Insulin resistance Fatiguability	14.4 ± 12.8 years [11]
Facioscapulohumeral muscular dystrophy type 1 and 2 (FSHD1/2)	Proximal weakness in the UL Proximal and distal weakness in the LL Wing scapula Facial weakness	Retinal vasculopathy/Coat syndrome Right bundle branch block High frequency hearing loss Pectus excavatus	Variable, from few years to several years [12]
Amyotrophic lateral sclerosis (ALS)	Bulbar onset: dysarthria, dysphagia Spinal onset: weakness in the upper or lower limbs, usually distal	Loss of weight Fatigue Shortness of breath Cognitive impairment	12 months [13]

CK, creatin kinase; DTRs, deep tendon reflexes; LL, lower limb; UL, upper limb.

**Table 2:** Main clinical findings and corresponding neuromuscular site of involvement, which can help to target the genetic analysis.

Main Neuromuscular Sign/Symptom	Possible/Probable Site of Lesion	Differential Diagnosis
Muscle weakness and stiffness, pseudobulbar signs, ↑↑ DTRs, Babinski and Hoffmann signs, clonus.	UMN	PLS ALS (UMN prevalent) HSP
Distal symmetric weakness, distal muscular atrophy, sensory and/or autonomic signs, ↓↓ DTRs, pes cavus, hammertoe deformities, leg atrophy. In general symptoms << signs.	Peripheral nerve	Genetic neuropathy (CMT)
Proximal muscle weakness and wasting, ↓↓ or absent DTRs, Gower’s sign, no sensory symptoms.	Skeletal muscle, LMN	Muscular dystrophies SMA type 3
Young age, proximal muscle weakness, facial weakness, diffuse wasting, ↓↓ or absent DTRs, Gower’s sign, bulbar signs, osteoskeletal deformities (pectus excavatus, scoliosis, tendon retractions, congenital hip dysplasia).	Skeletal muscle	CMs
Distal muscular weakness, grip myotonia, ↓↓ or absent DTRs, cataract, baldness, ptosis, bulbar signs.	Skeletal muscle	DM1
Proximal muscle weakness, normal or ↑↑ DTR, myotonia, myalgia, cataract	Skeletal muscle	DM2
Limb fasciculations associated with muscle weakness and/or atrophy, ↓↓ or absent DTRs, no sensory symptoms	LMN Peripheral nerve	ALS (LMN prevalent) Kennedy disaease (note that a sensory neuropathy could be also present) Pure motor neuropahy
Limb fasciculations associated with muscle weakness and/or atrophy, ↓↓ or absent DTRs, no sensory symptoms, bulbar signs	LMN	ALS (LMN prevalent) Kennedy disaease
Mixed LMN and UMN signs in the same myotome (e.g., muscle wasting, ↑↑ DTRs, fasciculations, muscle stiffness), bulbar signs	LMN and UMN	Classic ALS
Episodic weakness and/or paralysis	Skeletal muscle (ion channel)	Channelopathies
Fluctuating weakness with fatiguability, no sensory symptoms	Neuromuscular junction	Myasthenia gravis
Isolated “foot drop”	Peripheral nerve LMN Skeletal muscle	Genetic or acquired neuropathy ALS DM1 FSHD Distal myopathy
Isolated “drop head”	LMN Neuromuscular junction Skeletal muscle	ALS Miasthenia gravis Muscular dystrophies Metabolic myopathies
Isolated “bulbar signs”	LMN Neuromuscular junction	ALS Myasthenia gravis
Hypotonia and/or respiratory failure at birth	LMN Neuromuscular junction Skeletal muscle	SMA type 1 Congenital myasthenia CDM CMDs CMs Congenital myopathies Metabolic myopathy (Pompe disease)

ALS, amyotrophic lateral sclerosis; CDM, congenital DM; CMs, congenital myopathies; CMDs, congenital muscular dystrophies; CMT, Charcot–Marie–Tooth; DM1/2, myotonic dystrophy type 1 and 2; DTRs, deep tendon reflexes; FSHD, facioscapulohumeral dystrophy; HSP, hereditary spastic paraparesis; LL, lower limb; LMN, lower motor neuron; PLS, primary lateral sclerosis; SMA, spinal muscular atrophy; UMN, upper motor neuron; ↑↑, increased; ↓↓, decreased; <<, less than.
